# CD59, Disulphide‐Locked Human C9 and Horse C9 Inhibit Human Membrane Attack Complex Assembly by Similar Mechanisms

**DOI:** 10.1111/imm.70008

**Published:** 2025-06-16

**Authors:** Rebekah S. Cooke, Bradley A. Spicer, Richard A. Harrison, Michelle A. Dunstone, B. Paul Morgan, Wioleta M. Zelek

**Affiliations:** ^1^ Division of Infection and Immunity and UK Dementia Research Institute School of Medicine, Cardiff University Cardiff UK; ^2^ ARC Centre of Excellence in Advanced Molecular Imaging and Biomedicine Discovery Institute, Department of Biochemistry and Molecular Biology Monash University Melbourne Australia

**Keywords:** C9, CD59, complement, drugs, membrane attack complex

## Abstract

Five plasma proteins, C5b, C6, C7, C8 and C9, assemble in a step‐wise manner to form the membrane attack complex (MAC) which inserts into target cell membranes to cause lysis. The membrane regulator CD59 binds nascent C5b‐8, preventing C9 recruitment and polymerisation into the lytic pore. A disulphide‐locked C9 (‘C9lock’; C9_F262C/V405C_) lacked haemolytic activity in standard assays because the unfolding required for pore formation was prevented, while horse C9 (HoC9) lacked haemolytic activity suggested to be a consequence of species incompatibility in MAC assembly. In this study, we compared the impact of soluble CD59 (sCD59), C9lock and HoC9 on MAC assembly. C9lock and sCD59 were generated recombinantly, while HoC9 and human C9 (HuC9) were affinity‐purified from serum. Binding and haemolytic assays were used to identify and compare the modes of action of MAC binding and inhibition by sCD59, C9lock and HoC9. We show that sCD59, C9lock and HoC9 all inhibited human serum mediated haemolysis in both classical and alternative pathways. In reactive lysis assays, all three inhibitors bound immobilised C5b‐8 but not C5b‐7 intermediates on ELISA wells and gpE, and competitively blocked C9‐mediated lysis of gpE. Each of the inhibitors also bound mouse and rat C5b‐8 sites on gpE and blocked human C9‐mediated lysis. This work clarifies the functional differences between HoC9 and human C9 and highlights the mechanistic similarities of the diverse MAC inhibitors (C9lock, sCD59 and HoC9). These agents not only provide useful tools for analysis of MAC assembly but also signpost novel strategies for specific MAC inhibition in conditions where MAC formation contributes to pathology.

## Graphical abstract

The membrane attack complex (MAC) is formed by the stepwise assembly of five proteins: C5b, C6, C7, C8 and C9. MAC plays a key role in driving inflammation in numerous diseases. This study compares the inhibitory mechanisms of MAC assembly by soluble CD59 (sCD59), a disulphide‐locked C9 mutant (C9lock) and horse C9 (HoC9). All three inhibitors blocked MAC‐mediated haemolysis in the reactive lysis assay. This work elucidates the functional differences between HoC9 and human C9 while highlighting the shared mechanisms of action among the tested MAC inhibitors (C9lock, sCD59 and HoC9).

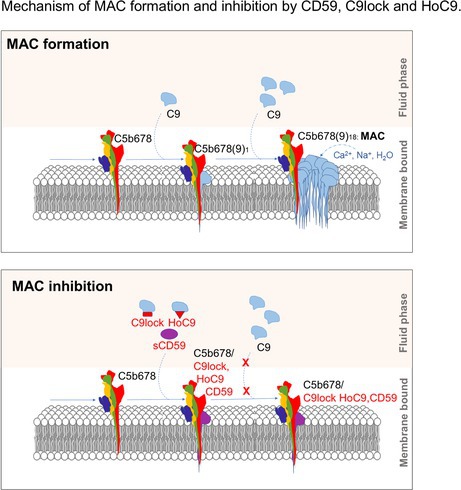

**Abbreviations**
Abs: absorbance
AP: alternative pathway
C5: complement component 5
C6: complement component 6
C7: complement component 7
C8: complement component 8
C9: complement component 9
CP: classical pathway
gpE: guinea pig erythrocytes
HBS: HEPES‐buffered saline
MAC: membrane attack complex
RbE: rabbit erythrocytes
RL: reactive lysis
RT: room temperature
sCD59: soluble CD59 protein
ShE: sheep erythrocytes
ShEA: antibody‐sensitised sheep erythrocytes

## 1 Introduction

The complement system is an essential component of the innate immune response, playing a critical role in defence against pathogens through a series of tightly regulated protein interactions. Activation of complement leads to the generation of several key effectors; opsonins (C3b/C4b), anaphylatoxins (C3a/C5a) and the membrane attack complex (MAC). The lytic MAC defends against pathogens, but in disease states, can also harm or kill self‐cells, propagating pathology. The formation of MAC involves the sequential assembly of five plasma proteins: C5, C6, C7, C8 and C9. This process begins with the convertase‐mediated cleavage of C5 into C5b; C5b captures C6 and C7 to form the C5b‐7 complex. This complex then binds adjacent membranes via a labile hydrophobic site, where it sequentially recruits C8 and up to 18 copies of C9, each component protein aligning and adopting an extended conformation that traverses the membrane [1, 2, 3]. The fully assembled MAC creates a lytic transmembrane pore that disrupts the integrity of the target cell membrane, leading to cell lysis [1, 2]. Regulation of MAC formation is crucial to prevent damage to host cells. On the cell membrane, CD59 is the principal MAC inhibitor; it binds the nascent C5b‐8 complex in a manner that prevents the first C9 from unfolding and inserting into the membrane, thus blocking recruitment of additional C9 molecules and pore formation [4, 5]. Further support for this mechanism of MAC inhibition was provided by modifying C9 to restrict unfolding; insertion of a disulphide lock in C9 (C9lock; F262C/V405C) prevented unfolding of the first trans‐membrane region on binding C5b‐8, blocking further C9 recruitment and pore assembly [3]. A related enigma is that of horse C9 (HoC9), structurally very similar to human C9 (HuC9 vs. HoC9; 77% sequence identity) but having little or no haemolytic activity in standard assays [6, 7]. Whether HoC9 functions as an inhibitor of MAC assembly is unclear.

To better understand how MAC assembly can be blocked and guide future development of MAC‐blocking drugs, we compared inhibitory mechanisms of CD59, C9lock and HoC9 in human serum and on human and rodent MAC intermediates assembled on cells or on an inert surface. We show that all three molecules bind the forming MAC at the C5b‐8 stage and prevent recruitment of C9 into the MAC, thereby blocking cell lysis. An understanding of the mechanisms by which these inhibitors block MAC pore formation provides valuable clues to the design of therapeutic approaches to MAC inhibition. This comparative analysis is particularly important for understanding mechanism and designing therapeutic strategies in the numerous diseases where MAC drives pathology, including systemic diseases such as paroxysmal nocturnal haemoglobinuria (PNH) and atypical haemolytic uremic syndrome (aHUS) and localised diseases such as neuromyelitis optica (NMO) and age‐related macular degeneration (AMD). The tools and assays presented will aid future design of MAC‐targeting therapies by providing an in‐depth understanding of MAC assembly and how this can be efficiently disrupted.

## 2 Materials and Methods

### 2.1 General

All chemicals, except where otherwise stated, were obtained from either Fisher Scientific UK (Loughborough, UK) or Merk (Sigma Aldrich; Gillingham, UK) and were of analytical grade. All tissue culture reagents and plastics were from Invitrogen Life Technologies (Paisley, UK). Horse serum, sheep (ShE), rabbit (rbE) and guinea pig erythrocytes (gpE) in Alsever's solution were from TCS Biosciences (Claydon, UK). Human, mouse and rat sera were prepared in house from freshly collected blood. For human and rat, blood was clotted at room temperature (RT) for 1 h (h), and then placed on ice for 2 h for clot retraction before centrifugation and harvesting of serum. For mouse, blood was placed on ice for 5 min (min) after harvest and clotted for 2 h on ice before serum harvest. Sera were stored in aliquots at −80° and not subjected to freeze‐thaw cycles.

### 2.2 Generation of Recombinant C9lock and Soluble CD59

A glycosylation‐mutant (N18Q) soluble form of CD59 (sCD59) was expressed essentially as described previously and produced in CHO cells [8]. sCD59 was purified from filtered culture supernatants by affinity chromatography on a 5 mL HiTrap column (GE Healthcare, #GE17‐0405‐01, Amersham, UK) on which 20 mg of anti‐CD59 monoclonal antibody (mAb) YTH53.1 had been immobilised. The supernatant was loaded, the column was washed extensively in PBS, bound protein was eluted in Glycine HCl pH 2.5, and dialysed into HBS. C9lock was expressed as reported by cloning the human C9 gene (P02748) with the F262C/V405C mutations into the pCDNA3.1 hygro+ vector (GenScript) followed by transient expression in Expi293F cells (ThermoFisher Scientific, #A14527, Waltham, NY, USA) [9]. Culture supernatants were harvested 7–14 days post transfection, filtered and diluted 1:1 with PBS pH 7.4 prior to affinity purification on a 5 mL HiTrap column on which 20 mg of anti‐C9 mAb B7 had been immobilised, washed, eluted and dialysed as above.

### 2.3 Purification of Horse, Human, Rat and Mouse C9

Native HuC9 was affinity purified from serum on a separate Hi Trap anti‐C9 mAb B7 column as described for C9lock. After demonstrating in ELISA and western blots that the anti‐C9 mAb B7 recognised HoC9 and rat C9 (data not shown), species‐dedicated anti‐C9 mAb B7 affinity columns were made specifically to purify the proteins from horse and rat serum using the same protocol. Mouse C9 was affinity purified from mouse serum using mAb C9‐6‐25‐5 anti‐mouse C9 (Hycult Biotech, #HM1134) immobilised on a 1 mL HiTrap column using the same protocol. Affinity purified HoC9 was subjected to gel filtration on a SD200 column (GE Healthcare, Superdex 200, 10/300 GL, #17‐5175‐01) to remove aggregates. Peak monomer fractions were pooled and stored at −20°C in HBS containing 20% glycerol. The protein concentrations were measured by absorbance at 280 nm (NanoDrop) and in the BCA assay (ThermoFisher, #23235).

### 2.4 Purification of Human C5b6, C7, C8

C7 and C8 were purified from human serum using immunoaffinity methods as previously described [10, 11, 12]. For the purification of C5b6, serum was first depleted of C7 by passage over a F10 anti‐C7 mAb column then fully activated by adding zymosan A (7 mg/mL; Pierce, #21327) and aggregated human IgG (50 μg/mL; in house) and incubating for 32 h at 37°C in a shaking water bath. The zymosan A was removed by centrifugation and the activated C7‐depeted serum applied to a RO7112689 anti‐C5 mAb column (gift of Roche); after washing, bound C5b6 was eluted using acetate buffer pH 5.5, dialysed, glycerol added to 20% and stored in aliquots at −20°C until use.

### 2.5 Characterisation of Purified Proteins

sCD59, C7, C8, HuC9, C9lock and HoC9 (5 μg) were resolved on 7.5% SDS–PAGE gels under reducing (R) and non‐reducing (NR) conditions. To detect protein bands, gels were stained with Coomassie Blue dye (ThermoFisher, #LC6065).

Dot‐blot analysis was performed to test the binding of the available anti‐C9 antibodies to HuC9 and HoC9 proteins. C9 proteins (2 μg in 5 μL PBS) were applied directly onto a nitrocellulose membrane and air‐dried. The membrane was blocked with 5% BSA in PBS containing 0.05% Tween 20 (PBST) for 1 h at 37°C to prevent non‐specific binding, then probed with either anti‐HuC9 mAbs (2 μg/mL) or polyclonal rabbit anti‐HuC9 (5 μg/mL) in blocking buffer and incubated for 1 h at 37°C. After washing with PBST, the membrane was incubated for 1 h at 37°C with either donkey anti‐mouse IgG‐HRP (for detection of anti‐HuC9 mAbs) or donkey anti‐rabbit IgG‐HRP (for detection of rabbit polyclonal anti‐HuC9; Jackson ImmunoResearch, #715‐035‐150, #711‐036‐152, respectively, Baltimore, USA) at a 1:10000 dilution in blocking buffer. The membrane was washed, and signals were visualised by applying a chemiluminescent reagent (Cytiva #RPN2106).

### 2.6 Assembling and Inhibiting MAC Intermediates on ELISA Plates

Human C5b6, C7 and C8 were immunoaffinity purified from serum as described above and the MAC intermediates built sequentially from these. Proteins were biotinylated using the EZ‐Link Sulfo‐NHS‐LC‐Biotin kit (ThermoFisher #A39257) according to manufacturer's instructions, dialysed to remove excess biotin and tested to confirm full retention of activity and immunogenicity in appropriate assays. Maxisorp plates (ThermoFisher #442404) were coated with anti‐C5 mAb RO7112689 [10] (5 μg/mL in bicarbonate buffer, pH 9.6, 50 μL/well) at 4°C overnight, wells blocked with 3% BSA in PBST (100 μL/well) for 1 h at 37°C and then washed with PBST. C5b6 was added (2 μg/mL in PBST 0.2% BSA (dilution buffer), 50 μL/well) and incubated for 1 h at 37°C. After washing, C7 was added (10 μg/mL in dilution buffer, 50 μL/well) and incubated for 1 h at 37°C, followed by another wash. C8 was added under the same conditions as C7. Binding of C5b6 and formation of the C5b‐7 and C5b‐8 complexes was confirmed in preliminary studies by detecting bound C5b6, C7 or C8 with specific mAbs. Binding of sCD59, HuC9, or HoC9 proteins to the immobilised MAC intermediates was tested by adding the biotinylated proteins in serial dilutions (700–0 nM) and incubating for 1 h at 37°C. After washing, wells were incubated with Streptavidin‐HRP (1:400 dilution, R&D Systems, #DY998, Minneapolis, USA) for 30 min at 37°C. After a final wash, plates were developed using o‐phenylenediamine dihydrochloride (OPD; SIGMAFAST TM; Sigma‐Aldrich, #P9187‐50SET), and absorbance was measured at 492 nm. Standard curves were automatically generated using GraphPad PRISM (GraphPad, La Jolla, CA, USA).

### 2.7 Assembling and Inhibiting MAC and MAC Intermediates on Erythrocyte Targets

The MAC inhibitory activity of C9lock, HoC9 and sCD59 in human and animal sera was first investigated using haemolysis assays. For the classical pathway (CP; CH50) assay, ShE were sensitised by incubation with rabbit anti‐ShE antiserum (#ORLC25, Siemens Amboceptor; Cruinn Diagnostics, Dublin, UK; ShEA), then suspended in HBS containing Ca^2+^ and Mg^2+^ at 2% (vol:vol) [11, 12, 13]; for measurement of CP activity in male mouse serum, ShEA were additionally sensitised with mouse anti‐rabbit IgG (25 μg/mL; Invitrogen, #3123) for 30 min at 37°C before washing and re‐suspending in HBS. Serum dilutions for each species were selected in preliminary experiments to give near complete haemolysis in the CP assay in the absence of test mAb, typically 2.5% for human serum, 25% for mouse serum (using the double‐sensitised cells as described above) and 2.5% for rat serum. A greater amount of mouse serum was used due to its markedly lower activity compared to human serum in haemolysis assays. A serial dilution series (1500–0 nM) of each potential blocker, C9lock, HoC9, sCD59 and a blocking anti‐C5 mAb (10B6) as positive control, was prepared in HBS and aliquoted in triplicate into a 96‐well round‐bottomed plate at 50 μL/well. Serum at the appropriate dilution and 2% ShEA (50 μL/well of each; double‐sensitised for mouse as above) was added, plates were incubated at 37°C for 30 min, centrifuged and haemoglobin in the supernatant was measured by absorbance at 405 nm. For alternative pathway (AP; AH50) haemolysis assay, unsensitised rabbit erythrocytes (RbE) were suspended in HBS containing 5 mM EGTA and 3 mM MgCl_2_ at 2% (vol:vol). Lytic serum dose was set and test blockers and control titrated for inhibition essentially as described for the CP assay. For each assay, percentage lysis was calculated according to: % Lysis = Absorbance (Abs) sample − Abs background)/(Abs max − Abs background) x 100%. GraphPad Prism (v. 5.0) was used for data analysis.

To test impact of the potential blockers on C5b6‐mediated reactive lysis (RL), guinea‐pig erythrocytes (gpE) at 2% in HBS (50 μL/well) were incubated (15 min at 37°C) with 50 μL/well C5b6 (0.125 μg/mL in HBS) in a 96‐well round‐bottom plate. C7 (in‐house, 0.7 μg/well) was added and incubated for 15 min at 37°C to form C5b‐7 sites, followed by C8 (1 μg/well) alone to form C5b‐8 sites or with C9 (1 μg/well) to complete MAC formation. Blockers were added at the C5b‐8 stage, incubated (15 min at 37°C) and either washed or not washed prior to addition of C9. Plates were incubated for 30 min at 37°C, centrifuged, supernatant harvested and % lysis calculated as above. In competition experiments, blocker and C9 were added simultaneously to C5b‐8‐gpE.

To test cross‐species activity, gpE bearing rat or mouse C5b‐8 sites were generated by incubation with C9‐depleted rat or mouse serum. After washing, HuC9 was added to develop lysis thus confirming C5b‐8 site formation and C9 species compatibility. C9lock, HoC9, or sCD59 at various doses were then pre‐incubated with C5b‐8‐bearing gpE prior to the addition of HuC9. In some experiments, C5b‐8‐bearing gpE were incubated for 15 min on ice with C9 (1 μg/well) to form C5b‐8C9_1_ intermediates then washed with ice‐cold HBS. These were incubated with blockers followed by C9 as described above and haemolysis measured.

### 2.8 In Vivo Testing of C9lock and sCD59

To test the capacity of C9lock and sCD59 to block mouse complement haemolytic activity in vivo, groups of three wild‐type (WT) mice (C57BL/6J, bred in‐house) were administered the respective proteins or BB5.1 anti‐mouse C5 inhibitory mAb (1 mg in PBS; 40 mg/kg dose), or vehicle (PBS alone) by intraperitoneal (IP) injection. Blood was collected 2 h after IP injection for measurement of haemolytic activity as described above.

### 2.9 Statistical Analysis

All data analyses were conducted using GraphPad Prism software (version 5.0, San Diego, CA). Statistical significance between groups was determined using an unpaired *t*‐test. A *p*‐value of less than 0.05 was considered statistically significant. IC_50_ values were calculated automatically by the software. Error bars in all figures represent the mean ± standard error of triplicate measurements unless stated otherwise. Statistical significance was obtained by unpaired *t*‐test, and *p* < 0.05 was considered significant; significant differences and *p*‐values, mean and SD are shown in the figures.

## 3 Results

### 3.1 Affinity Chromatography Yields Pure Recombinant C9lock and sCD59 and Native Human C9, C8, C7, C5b6 and Horse C9

**FIGURE 1:**
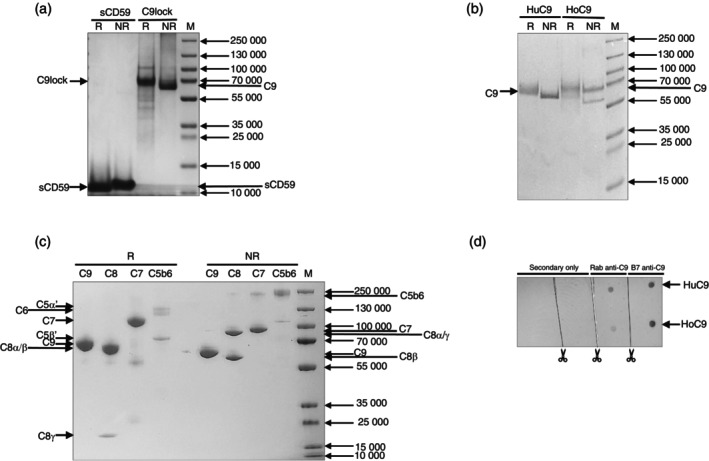
Purity of the proteins confirmed by SDS–PAGE of (a) purified recombinant human C9lock and sCD59 (N18Q) (4 μg) and (b) plasma‐purified horse and human C9 on 7.5% PAGE. Proteins were run non‐reduced (NR) or reduced with 5%β‐mercaptoethanol (R), proteins and gels stained with Coomassie Blue. Recombinant C9lock, native NR C9lock, Horse C9 (HoC9) and Human C9 (HuC9) all had Mrs in the range 65–75 kDa; unglycosylated sCD59 had a Mr ~12 KDa. (c) C5b6; C5b ~200 kDa band and ~100 kDa band corresponds to C6 (NR). C5α′ chain; ~100 kDa and C5β; chain ~75 kDa, C6; ~100 kDa (R). C7 ~95 kDa (NR), and ~100 kDa (R), C8; ~90 kDa C8α/γ and ~65 kDa C8β (NR), ~70 kDa C8α/C8β and ~20 kDa C8γ on R. (d) Dot‐blot analysis of HuC9 and HoC9 to confirm mAb B7 specificity. The intensity of the dots indicates the presence of C9 protein detected either with mAb B7 anti‐C9 or rabbit anti‐C9.

To obtain purified proteins we performed affinity chromatography. To identify horse C9‐specific mAb, we performed a dot‐blot analysis. Of several anti‐HuC9 mAbs tested, only one, mAb B7, detected HoC9 in a dot blot assay (Figure 1**c**). Having demonstrated binding to HoC9, HuC9, HoC9 and C9lock were each affinity purified in a single step on individual B7 anti‐C9 mAb HiTrap columns, the native proteins from their respective sera and recombinant C9lock from culture supernatants. Recombinant glycosylation (N18Q) mutant sCD59 was affinity purified from culture supernatants on a YTH53.1 anti‐CD59 antibody HiTrap column. To assess purity SDS‐PAGE analysis were performed; gels of the purified proteins demonstrated purity; sCD59 showed a single strong band at 12 kDa non‐reduced and 11 kDa reduced, C9lock and native HuC9 both ran as a single major band at 65 kDa non‐reduced and 68 kDa reduced, while HoC9 ran as a single major band at 68 kDa non‐reduced and a major band at 75 kDa with a minor band at ~60 kDa reduced, likely a HoC9 degradation product (Figure 1a,b). C5b6 resolved under non‐reducing conditions as two bands, a ~200 kDa band corresponding to C5b and a ~100 kDa band corresponding to C6, and in reducing conditions, C6 and C5α′ chain at ~100 kDa and C5β chain at ~75 kDa. C7 was a single major band at ~95 kDa non‐reduced and ~100 kDa reduced. C8 as ~90 kDa C8αγ and 65 kDa C8β bands non‐reduced and ~70 kDa C8α/C8β and ~20 kDa C8γ reduced (Figure 1c). Yields of HuC9 and HoC9 proteins were similar, between 5 and 8 mg from 100 mL serum. Yields of the recombinant proteins varied from batch to batch but were typically 1–2 mg sCD59 from a 50 mL CHO supernatant batch and 3–5 mg C9lock from a 50 mL Expi293F supernatant batch.

### 3.2 C9lock, HoC9 and sCD59 All Inhibit Haemolysis in Classical and Alternative Pathway Haemolysis Assays and Reactive Lysis

**FIGURE 2:**
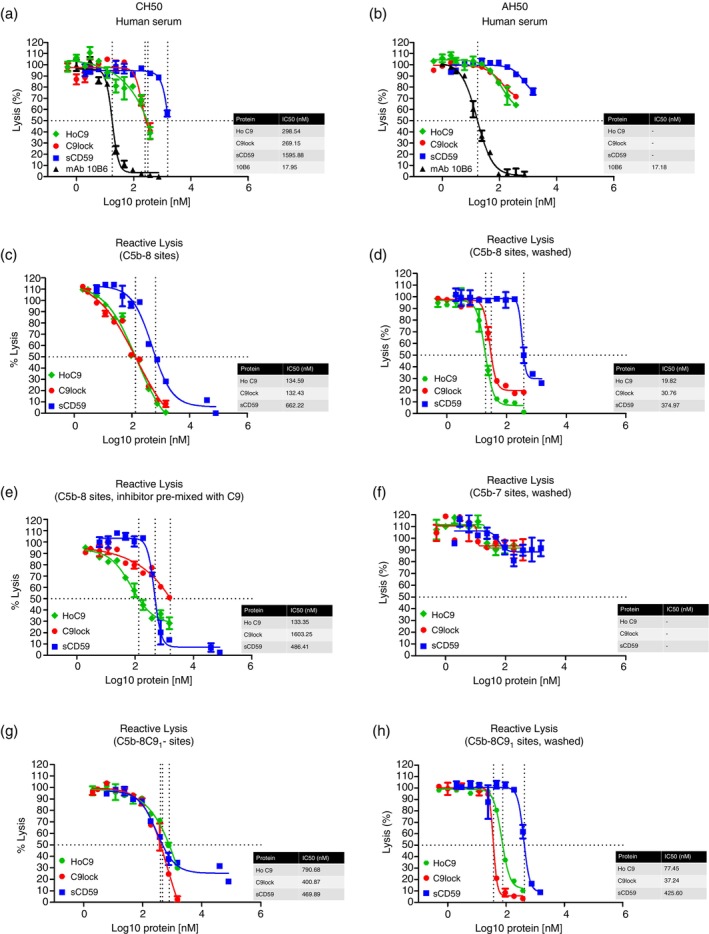
Haemolytic assays comparing C9lock, HoC9 and sCD59 activity in human. The proteins were tested for blocking activity in classical pathway (CH50) against in house blocking mAb 10B6 anti‐C5 showing the moderate ability of the inhibitors to compete with HuC9. (a) and alternative pathway (AH50) (b). C9lock and HoC9 (with or without C9) and sCD59 were compared in reactive lysis using purified components confirming their ability to inhibit MAC formation in this controlled purified system (c, d). Inhibitors were premixed with HuC9 to test whether they compete for the C5b‐8 binding site (e). Point of action was explored by comparing effects of C9lock and sCD59 on C5b‐7, C5b‐8 and C5b‐9_1_ sites with or without washing (f–h). IC_50_ values were calculated in nM from the logged values showed on the graphs.

To assess functional activity, we tested all three inhibitors in complement functional assays; C9lock, HoC9 and sCD59 all dose‐dependently inhibited haemolysis in classical (IC_50_ = 269.15 nM, 298.54 nM and 1595.88 nM, respectively; Figure 2a) and, to a lesser extent, alternative (Figure 2b) pathway assays. For each test protein, inhibition was weak in comparison to the positive control C5‐blocking mAb 10B6, requiring ~10–100‐fold more agent (on a molar basis) to achieve 50% inhibition of lysis in the classical pathway. As expected, native HuC9 had no impact on haemolysis in either assay (not shown).

To confirm that the observed inhibitory activity for all agents was at the final steps of MAC assembly, C5b‐8 sites were assembled on gpE by sequential addition of C5b6, C7, and C8. Each of the three test proteins, when pre‐incubated with gpE‐C5b‐8, dose‐dependently inhibited lysis when HuC9 was subsequently added (HoC9 IC_50_ = 134.59 nM; C9lock IC_50_ = 132.43 nM; sCD59 IC_50_ = 662.22 nM; Figure 2c). To determine whether these inhibitors firmly bound C5b‐8, we added a wash step after incubation with the test proteins and before adding HuC9; each of the three test proteins inhibited in the wash assays, demonstrating that they firmly bound the C5b‐8 site. Each agent showed better inhibition in wash assays (HoC9 IC_50_ = 19.82 nM; C9lock IC_50_ = 30.76 nM; sCD59 IC_50_ = 374.97 nM; Figure 2d), suggesting that fluid phase MAC precursors competed in the assay. To assess whether HoC9 competes with HuC9, the test proteins were added simultaneously with HuC9 (47 nM each); all retained dose‐dependent inhibition, although both C9lock and HoC9 were less effective compared to sCD59 (HoC9 IC_50_ = 133.35 nM; C9lock IC_50_ = 1603.25 nM; sCD59 IC_50_ = 486.41 nM; Figure 2e). To test binding to other MAC intermediates, the binding experiments were repeated using C5b‐7 sites and C5b‐8C9_1_ sites. Washing after incubation of gpE‐C5b‐7 with test agents and prior to the addition of C8 and C9 completely ablated inhibition for all, demonstrating that none bound the C5b‐7 complex (Figure 2f). All test agents efficiently inhibited C9‐mediated lysis of gpE‐C5b‐8C9_1_ intermediates (HoC9 IC_50_ = 790.68 nM; C9lock IC_50_ = 400.87 nM; sCD59 IC_50_ = 469.89 nM; Figure 2g), further improved by a wash step for HoC9 and C9lock (HoC9 IC_50_ = 77.45 nM; C9lock IC_50_ = 37.24 nM; sCD59 IC_50_ = 425.60 nM; Figure 2h), demonstrating strong binding to the forming MAC even after capture of the first C9.

### 3.3 HoC9, C9lock and sCD59 Inhibit MAC Assembly in Rodents

**FIGURE 3:**
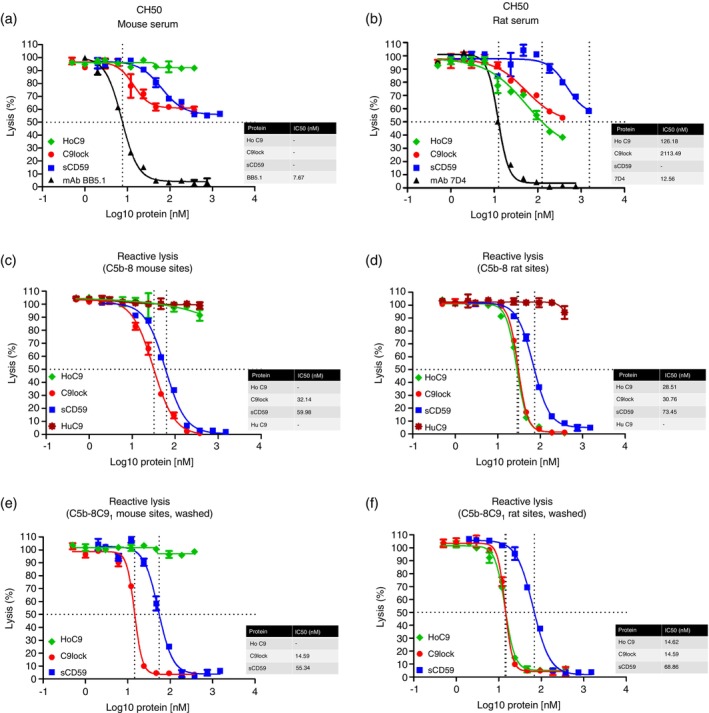
Haemolysis assays demonstrating inhibition of lysis by C9lock, HoC9 and sCD59 on rodent sera. All inhibitors show dose–response curves demonstrating their blocking activity in the classical pathway (CH50) using rat serum, confirming cross‐species‐specific activity (b). HoC9 did not prevent mouse serum‐mediated lysis (a). To further confirm inhibition at the C5b‐8 stage, reactive lysis assays were performed. Reactive lysis assays utilising C5b‐8 sites from rats or mice confirmed the inhibition by sCD59, HoC9 and C9lock in rat serum, but not by HoC9 in mouse serum (c, d). To assess ability of the inhibitors to bind C5b‐9_1_ sites reactive lysis assays were used. Comparison the effects of C9lock, HoC9 and sCD59 on C5b‐9_1_ sites, using mouse or rat serum to confirm cross‐species reactivity of CD59 and C9lock with both rat and mouse, and HoC9 with rat only (e, f). IC_50_ values were automatically calculated in nM from the logged values included on the graphs.

We next tested whether HoC9, C9lock and sCD59 inhibited haemolysis in classical pathway assays using mouse and rat serum. In mouse serum, C9lock and sCD59 both dose‐dependently inhibited haemolysis, albeit poorly in comparison with the mouse C5 blocking mAb BB5.1, while HoC9 showed no inhibition of lysis in mouse serum (Figure 3a). In rat serum, all three test agents dose‐dependently inhibited lysis, though weakly compared to the rat C5 blocking mAb 7D4 (HoC9 IC_50_ = 126.18 nM; C9lock IC_50_ = 2113.49 nM; Figure 3b). To confirm that inhibition occurred at the C5b‐8 stage in these other species, gpE bearing either mouse or rat C5b‐8 sites were generated using C9‐deficient mouse or rat serum. Addition of HuC9 to these washed intermediates caused complete lysis (C9lock IC_50_ = 32.14 nM; sCD59 IC_50_ = 59.98 nM and HoC9 IC_50_ = 28.51 nM; C9lock IC_50_ = 30.76 nM; sCD59 IC_50_ = 73.45 nM; Figure 3c,d). For mouse, preincubation of gpEC5b‐8 with C9lock or sCD59 dose‐dependently inhibited HuC9‐mediated lysis while HoC9 caused no inhibition (Figure 3c). For rat, preincubation of gpEC5b‐8 with each of the three agents efficiently inhibited lysis (Figure 3d). When HuC9 was added to mouse or rat C5b‐8 sites on ice to make C5b‐8C9_1_ sites as described above, the same inhibitory profile was seen (C9lock IC_50_ = 14.59 nM; sCD59 IC_50_ = 55.34 nM and HoC9 IC_50_ = 14.62 nM; C9lock IC_50_ = 14.59 nM; sCD59 IC_50_ = 68.86 nM; Figure 3e,f).

### 3.4 Binding of C9lock, HoC9 and sCD59 to Human C5b‐8 Is Recapitulated in ELISA

**FIGURE 4:**
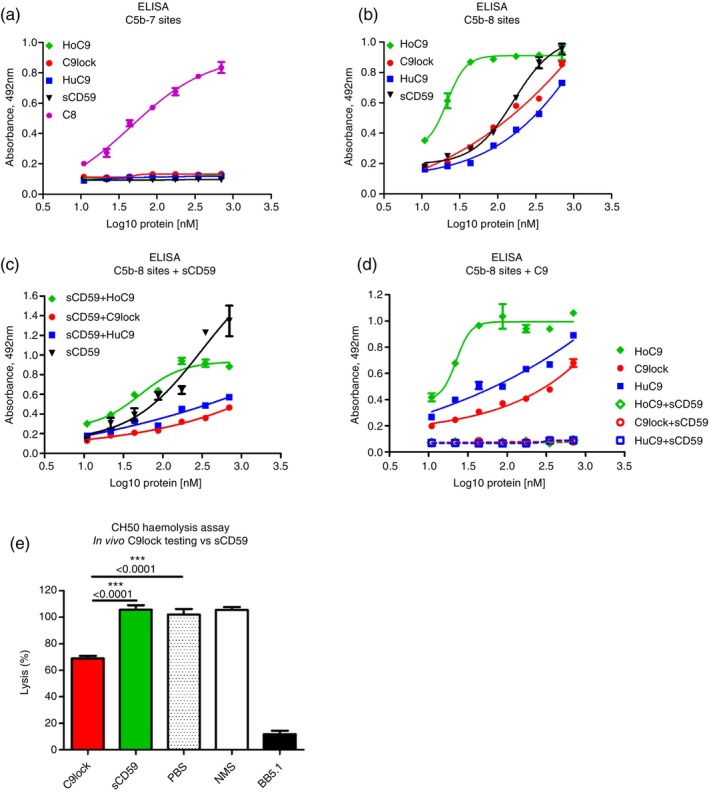
Binding of C9lock, HoC9 and sCD59 to human C5b‐8 measured by ELISA. C5b6 was immobilised on plates coated with anti‐C5 mAb RO7112689, C7 and C8 were added to form C5b‐7 and C5b‐8 sites to investigate the binding of sCD59, C9lock, HoC9 and HuC9 to ether C5b‐7 and C5b‐8 complexes. (a, b) Dose–response curves showing the binding of HoC9, HuC9, C9lock and sCD59 to the C5b‐8 complex, and the lack of binding to the C5b‐7 complex. When sCD59 was added first to the C5b‐8 complex, followed by biotinylated HuC9, HoC9 and C9lock. (c), binding was partialy inhibited. When either HoC9, HuC9 or C9lock were added first to the C5b‐8 sites, followed by biotinylated sCD59, binding of sCD59 to C5b‐8 complex was completely inhibited (d). In vivo testing was performed to investigate the ability of C9lock and sCD59 to inhibit haemolytic activity in mice (e). Mice (female, *n* = 3) were injected IP with either C9lock or sCD59 at a dose of 40 mg/kg. Blood was collected 2 h post IP injection and sera tested for complement‐mediated lysis using a CH50 assay. C9lock significantly reduced haemolysis by an average of 36.7%, while sCD59 was ineffective, showing lysis levels comparable to PBS‐treated controls. BB5.1 anti‐mouse C5 administered at the same dose, used as a positive control, caused complete lytic inhibition. Normal mouse serum (NMS) was included as an additional full lysis control.

To further investigate the binding interactions of these inhibitors with MAC intermediates, we performed ELISA assays. Human C5b6 was captured through a C5b‐binding mAb on ELISA plates, enabling the sequential assembly of C5b‐7 and C5b‐8 sites. C9lock, HoC9, HuC9 and sCD59 all showed no binding to C5b‐7 sites assembled on the wells, although C8 strongly bound the complex (Figure 4a). In contrast, all four test agents bound strongly to C5b‐8 sites, HoC9 in particular displaying strong, saturable binding (Figure 4b). Preincubation of the C5b‐8 sites with an excess of sCD59 partially inhibited the subsequent binding of HoC9, HuC9 and C9lock to C5b‐8 (Figure 4c). In contrast, preincubation of the C5b‐8 sites with either HoC9, HuC9 or C9lock completely prevented subsequent binding of sCD59 to the C5b‐8 complex (Figure 4d).

### 3.5 C9lock Reduced MAC Lytic Activity In Vivo, While sCD59 Did Not

To assess the capacity of C9lock and sCD59 to inhibit complement in vivo, groups of mice were injected IP with C9lock, sCD59 (each at 40 mg/kg) or vehicle; blood was harvested 2 h post injection. In the CH50 haemolysis assay, sera of animals treated with C9lock showed an average reduction in lytic activity of 33.02% compared to vehicle controls while sCD59 caused no inhibition (Figure 4e). As a positive control, mice were treated with the same dose of a potent mouse C5 blocker, mAb BB5.1; haemolysis was ablated at 2 h (Figure 4e).

## 4 Discussion

Accumulating evidence implicates the MAC in pathology in various diseases by contributing to inflammation, activating downstream signalling pathways and/or triggering immune responses [1, 12, 14, 15, 16]. In recent years, structural studies using cryo‐EM and atomic force microscopy have contributed to the understanding of MAC assembly. These studies identified the binding of C5b‐8 to C9 as a ‘kinetic bottleneck’, the slowest step in MAC assembly, presenting an optimal opportunity for physiological inhibition and therapeutic intervention [1, 2, 3, 17]. The natural inhibitor of this stage of MAC assembly is CD59, a GPI‐linked membrane glycoprotein that locks into the forming MAC at the C5b‐8 stage and inhibits further assembly by preventing C9 unfolding and inserting into the membrane [4, 5, 18, 19]. Early studies explored whether soluble CD59 (sCD59) could inhibit MAC assembly; although capable of inhibition in reactive lysis systems, the presence of serum markedly reduced inhibitory activity [20, 21, 22], suggesting that sCD59 was unlikely to be useful as a therapeutic complement inhibitor. These early studies also demonstrated that sCD59 and C9 competed for overlapping epitopes on C8 in the C5b‐8 complex, raising the possibility that modified C9 analogues might block MAC formation [4, 18, 19]. The observation that C9 isolated from horse serum (HoC9) bound human C5b‐8 sites but did not cause haemolysis supported this possibility [6], while the demonstration that a disulphide‐locked C9 (C9lock), incapable of unfolding for membrane insertion, blocked further C9 recruitment into the MAC further reinforced the concept [3].

We here compared the MAC inhibitory properties of these three putative inhibitors; all dose‐dependently inhibited haemolysis in classical and alternative pathway assays in human serum, albeit weakly in comparison with a potent C5‐blocking mAb 10B6; C9lock and HoC9 showed similar inhibition profiles, and both were more potent inhibitors than sCD59 in these whole serum assays. To confirm that all three putative inhibitors acted at the final stage of MAC assembly and test whether this represented a potential therapeutic target, we used preformed C5b‐7 and C5b‐8 sites and reactive lysis assays to investigate and compare the mechanisms of MAC inhibition utilised by sCD59, C9lock and HoC9. The mechanism by which CD59 inhibits MAC formation is well understood, first described in 1990 and recently confirmed by cryo‐EM [4, 5, 17, 18, 19]. CD59 binds C8α in the C5b‐8 complex, allowing the first C9 to bind but blocking its unfolding and insertion into the membrane, thus preventing the recruitment of additional copies of C9. The mechanism of action of C9lock is similar in that it binds C5b‐8, but the unfolding of the TMH1 domain upon binding is prevented, blocking the sequential recruitment of C9 monomers to the growing MAC pore [3, 6]. The precise mechanism by which HoC9 inhibits MAC assembly is unclear.

In our study, C9lock, HoC9 and sCD59 all bound C5b‐8 but not C5b‐7 complexes, assembled on erythrocyte targets or in ELISA wells, and effectively prevented the binding of native human C9 to the C5b‐8 complex. Washing of human C5b‐8‐bearing cells after the addition of inhibitor had no impact on inhibition by C9lock or HoC9, demonstrating strong binding to C5b‐8; in contrast, inhibition by sCD59 was reduced but not eliminated by washing, suggesting weaker binding. When tested on C5b‐8C9_1_ sites, where the first C9 has bound but not unfolded, C9lock, HoC9 and sCD59 all retained the capacity to inhibit even after washing. There are three possible explanations for this unexpected finding: first, that the inhibitors can still bind C5b‐8 even when C9_1_ is bound; second, that they competitively displace C9_1_ from C5b‐8 to enable binding; third, that they bind the partially unfolded C9_1_ to block further C9 recruitment. The first, though possible for sCD59, is unlikely for C9lock and HoC9 as they likely bind identical sites in C5b‐8; for the same reason, the latter two mechanisms are unlikely for sCD59 but could explain inhibition by C9lock and HoC9.

To further explore this we performed competition studies testing whether the agents competed for binding sites on C5b‐8. Pre‐binding of C9lock or HoC9 to the C5b‐8 complex completely blocked subsequent binding of sCD59, but in the reverse situation, when sCD59 was pre‐bound to C5b‐8, binding of C9lock or HoC9 was reduced but not ablated. These data suggest that the binding sites for sCD59 and the C9‐like blockers partially overlap; binding of the large C9 analogues completely block the binding sites while binding of the small, compact CD59 molecule permits subsequent binding of C9 analogues. The findings imply that all three inhibitors act in similar ways, sCD59 by binding C5b‐8 and sterically restricting C9 unfolding, C9lock because it is unable to unfold after binding C5b‐8 sites, while HoC9 likely inhibits in a manner similar to C9lock, failing properly to unfold after binding C5b‐8 and hence blocking recruitment of native C9 to the forming MAC. Structural analyses of these complexes would add further clarity to their modes of action.

Testing in vivo in mice in a single‐dose, single‐timepoint study demonstrated that C9lock significantly reduced haemolytic, albeit incompletely and weakly compared to a C5‐blocking mAb, while sCD59 caused no inhibition in vivo as has been previously reported [19]. Further dosing and timecourse studies are required to fully assess the potential of C9lock for therapeutic inhibition of complement. Of note, HoC9 was not tested in vivo because it showed no inhibition of mouse complement in vitro.

The pathological involvement of the MAC is well established in numerous diseases. By elucidating the mechanisms underlying MAC inhibition, this study offers critical insights that may inform the design of effective therapeutic strategies. Moreover, the assays and tools described lay a foundation for the development of interventions capable of efficiently disrupting MAC assembly across many diseases. For further testing of these agents, function in rodents is an advantage, opening up in vivo experiments. C9lock, HoC9 and sCD59 all inhibited haemolysis in rat serum and bound rat C5b‐8 sites, effectively preventing lysis by human C9. In mouse, while C9lock and sCD59 worked as in rat, HoC9 showed no inhibition of mouse serum haemolytic activity and no binding to mouse C5b‐8 sites, demonstrating an important species difference.

Several agents that block MAC assembly by C5 blockade are already in the clinic for treatment of complement‐driven diseases [23]; these agents prevent C5 cleavage, thus blocking C5a generation and MAC assembly. The proteins tested here block downstream of C5 cleavage to specifically block MAC assembly. Whether any of these proteins could be exploited therapeutically as MAC inhibitors remains to be explored. Of note, sCD59, expressed locally in the retina using an adenoviral delivery vector, was shown to inhibit retinal damage in a mouse macular degeneration model, suggesting that local inhibition of MAC has potential [24]. Indeed, a modified version of this gene delivery approach has progressed to phase 2b clinical trials in human macular disease (NCT05811351), demonstrating that, at least in the local environment, sCD59 inhibits MAC‐mediated damage. We show here that C9lock and HoC9 inhibit at the same stage of MAC assembly as sCD59 and with higher activity. We suggest that human C9, modified either as in C9lock or to mimic the behaviour of HoC9, might prove effective as an approach to MAC inhibition in disease.

## Author Contributions

W.M.Z. and R.S.C. performed all the laboratory analyses. W.M.Z. wrote the first draft of the manuscript. B.A.S. and M.A.D. generated the C9lock vector. W.M.Z., R.A.H. and B.P.M. conceived and planned the study and oversaw the data handling and manuscript preparation. All authors contributed to the article and approved the submitted version.

## Conflicts of Interest

The authors declare no conflicts of interest.

## Data Availability

The data that support the findings of this study are available from the corresponding author upon reasonable request.
